# Female genital mutilation and urinary incontinence: an analytical comparison with Sudan's prevalent demography

**DOI:** 10.1590/1806-9282.20231663

**Published:** 2024-08-16

**Authors:** Mustafa Cengiz Dura, Hilal Aktürk, Salih Mahmoud Abaker Salih, Özgür Aslan, Metehan Hergüner, Murat Ekin

**Affiliations:** 1 The University of Health Science, Bakırköy Dr. Sadi Konuk Education and Research Hospital - İstanbul, Turkey.; 2 Sudan Nyala Turkish Hospital - Khartoum, Sudan.; 3 Muş State Hospital - Muş, Turkey.

**Keywords:** Genital mutilation, female, Infibulation, Sudan, Urinary incontinence

## Abstract

**OBJECTIVE:**

Female genital mutilation/cutting impacts over 200 million women globally and is linked to obstetric complications as well as long-term urogynecological and psychosexual issues that are frequently overlooked and inadequately addressed. This study aimed to assess the impact of female genital mutilation/cutting on urinary incontinence.

**METHODS:**

This cross-sectional study was conducted in the gynecology department of the Research Hospital located in the Nyala rural region of Sudan. The participants were interviewed to gather socio-demographic and background information. In addition, they received a thorough gynecological examination to evaluate the presence and type of female genital mutilation/cutting. The Incontinence Impact Questionnaire and the Urogenital Distress Inventory were applied to the group with female genital mutilation/cutting and the control group without female genital mutilation/cutting to evaluate urinary incontinence and related discomfort. Subsequently, the scores of both participant groups were compared.

**RESULTS:**

The study compared age, weight, height, BMI, gravida, parity, and sexual intercourse averages between groups. The mean Urogenital Distress Inventory-6 and Incontinence Impact Questionnaire-7 scores of individuals who underwent mutilation were higher than those of individuals who did not undergo mutilation (p<0.001). Notably, participants subjected to infibulation exhibited significantly higher average scores on both measures in contrast with the other groups (p<0.001).

**CONCLUSION:**

A higher proportion of mutilated participants, specifically those with infibulation, are afflicted with symptoms of incontinence.

## INTRODUCTION

The cultural practice of female genital mutilation (FGM), also known as female genital mutilation/cutting (FGM/C), has been deeply rooted in the cultural customs of certain regions in Sudan for many centuries^1^. FGM/C is a cultural practice that is also widespread in Africa and Asia. It is usually performed by traditional practitioners and has a significant impact on more than 200 million women globally^2,3^. FGM/C involves the partial or total removal of the external female genitalia often performed without medical supervision or proper hygiene.

The World Health Organization (WHO) classifies the act of cutting the female genital region as "mutilation"^4^. WHO categorized FGM into four distinct kinds. Type I is also referred to as clitoridectomy. Type II entails the partial or total excision of the labia minora and majora, as well as the removal of the clitoris. Type III, which is often referred to as infibulation, entails the surgical removal of a part or the totality of the external genitalia, followed by the surgical approximation of the remaining labia majora. Type IV comprises a range of injuries to the female vaginal organs^5^.

While some argue that FGM is a customary practice or an important rite of passage. Its potential harmful health effects have gained global recognition^6^. Among these, health risks is an increased incidence of urinary incontinence (UI) among women who have undergone the procedure.

UI refers to the involuntary release of urine, which can significantly affect an individual's quality of life, self-esteem, and overall well-being. The severity and frequency of this condition can vary from occasional minor leakage to more frequent and debilitating symptoms^7^.

While the physical and psychological health effects of FGM have been well examined, the urinary issues resulting from the procedure have not received adequate attention in the existing literature. Abdulcadir et al.'s study conducted in Sudan demonstrated a significant association between FGM and UI among women^8^.

Similarly, Dirie and Lindmarks's study done in Somalia revealed a high prevalence rate of 80% for UI among women who had undergone FGM^8^.

This study aims to conduct a comprehensive examination of the prevalence of UI among Indigenous Sudanese women who have experienced FGM. This study also aims to examine differences in the characteristics and severity of UI according to different classifications of FGM.

## METHODS

This study used a cross-sectional design to evaluate the effect of mutilation on UI in an endemic Sudanese female population. A total of 307 people participated in the study. The research was conducted in collaboration with healthcare professionals, researchers, and local communities within Sudan. Approval was granted by the institutional ethics committee (NSTH.03/903.07.03/689, date: 01.03.2021). The study was conducted in accordance with the Declaration of Helsinki and followed the ethical standards of Turkey.

A representative sample of women who had undergone FGM was selected using a multi-stage sampling technique. Efforts were made to ensure diversity in age groups, marriage status, educational status, circumcise type, and menopausal status to capture a comprehensive picture of the endemic population. Informed consent was obtained from all participants before their inclusion in the study. The exclusion criteria were the presence of a urinary tract infection identified through clinical examination or urine analysis, confirmed renal disease, cervicitis and/or vaginitis, diabetes diagnosis, vaginal and/or urethral surgery, and rejection to participate in the study.

Data collection was carried out through face-to-face interviews using structured questionnaires. The questionnaires were designed based on established instruments used in previous studies exploring the impact of female genital circumcision on UI. We use the Urogenital Distress Inventory (UDI)-6 and the Incontinence Impact Questionnaire (IIQ)-7. Both questionnaires underwent validation in the Arabic language^9,10^.

The UDI-6 is a validated health-related quality-of-life (HRQOL) tool that assesses the level of distress caused by three categories of urine symptoms: irritative, obstructive/discomfort, and stress^11^. The total score is from 0 to 100^12^. The IIQ-7 is a psychometric questionnaire specifically designed to assess UI. This questionnaire evaluates the psychological and social effects of UI in women. The overall score spans from 0 to 100^13^.

The UDI-6 Total Score of 33.33 and IIQ-7 Total Score of 9.52 were identified as the most effective thresholds for differentiating between women with symptoms and those without symptoms^14^.

The questionnaires were administered to the patients by the local gynecologist. Participants were asked about the frequency, amount, and impact of urine leakage, as well as any associated physical or emotional discomfort.

In the analysis of the data, the SPSS version 25.0 program was used. The suitability of the variables for normal distribution was examined with histogram graphs and the Kolmogorov-Smirnov test. While presenting descriptive analyses, mean, standard deviation, median, and minimum-maximum values were used. The 2×2 eyes were compared with the Pearson chi-square test. Variables not showing normal distribution (nonparametric) were evaluated between groups using the Mann-Whitney U test, and among more than two groups using the Kruskal-Wallis test. Situations, where the p-value is below 0.05, were considered statistically significant results.

## RESULTS

The study included a total of 307 participants. The mutilation group consisted of 161 (52.44%), whereas the control group had 146 (47.56%) participants. The average UDI-6 and IIQ-7 scores of circumcised individuals were higher than those of uncircumcised individuals.

The marital status, education, and menopausal status of the circumcised and uncircumcised individuals were compared in this study. The findings indicate that there were no significant differences between the groups. The study compared several factors including age, weight, height, BMI, gravida, parity, and sexual intercourse averages between the two groups. The results indicated that unmutilated participants had a higher average sexual intercourse frequency compared with circumcised individuals (p<0.001).

The UDI-6 and IIQ-7 averages were compared between the groups. The average UDI-6 and IIQ-7 scores of circumcised individuals were higher than those of uncircumcised individuals (Table 1).

**Table 1:** The average UDI-6 and IIQ-7 scores of individuals.

	Group				p
	Mutilated		Unmutilated		
	Mean±SD	Median (min-max)	Mean±SD	Median (min-max)	
UDI-6	22.27±28.13	5.55 (0-88.8)	15.05±22.67	0 (0-83.25)	**0.036**
IIQ-7	18.21±25.28	4.75 (0-91.84)	10.38±18.49	0 (0-80.87)	**0.015**

Mann-Whitney U test.

Statistically significant values are indicated in bold.

A comparison was made between different mutilation types based on their associated UDI 6 and IIQ 7 scores (Table 2). Notably, participants subjected to infibulation exhibited significantly higher average scores on both measures in contrast with the other groups (p<0.001). Evidently, this pattern persisted across both measures (UDI 6 and IIQ 7) illustrating a distinct disparity between those who received infibulation versus those who did not (p<0.001).

**Table 2:** Comparison of UDI-6 and IIQ-7 scores by circumcision type.

	Circumcised type								p
	Uncircumcised		Clitoridectomy		Excision		Infibulation		
	Mean±SD	Median (min-max)	Mean±SD	Median (min-max)	Mean±SD	Median (min-max)	Mean±SD	Median (min-max)	
UDI-6	15.05±22.67	0 (0-83.25)	3.77±11.3	0 (0-49.95)	13.23±20.28	0 (0-77.7)	40.08±30.96	44.4 (0-88.8)	**<0.001**
IIQ-7	10.38±18.49	0 (0-80.87)	2.65±8.73	0 (0-42.42)	9.5±17.44	0 (0-76.11)	34.49±28.46	38.05 (0-91.84)	**<0.001**

Kruskal-Wallis test.

Statistically significant values are indicated in bold.

The hierarchical regression results for UDI-6 and IIQ-7 scores are detailed in Table 3.

**Table 3:** Hierarchical regression results for UDI-6 and IIQ-7.

UDI-6								
		B	Std. error	Beta	t	95%CI for B		p
Model 1						LL	UL	
	Age	0.696	0.097	0.391	7.199	-19.319	38.276	0.518
	BMI	-0.456	0.426	-0.055	-1.071	0.506	0.887	<0.001
	Gravida	1.827	1.150	0.138	1.588	-1.294	0.382	0.285
	Parity	1.781	1.890	0.082	0.943	-0.436	4.090	0.113
	Sexual intercourse	-0.757	0.608	-0.064	-1.245	-1.938	5.501	0.347
Model 2						-1.955	0.440	0.214
	Age	0.694	0.096	0.390	7.242	0.505	0.883	<0.001
	BMI	-0.439	0.422	-0.053	-1.039	-1.269	0.392	0.300
	Gravida	1.723	1.140	0.130	1.511	-0.521	3.966	0.132
	Parity	1.842	1.873	0.084	0.984	-1.843	5.527	0.326
	Sexual intercourse	-0.297	0.628	-0.025	-0.473	-1.534	0.939	0.637
	Group*	6.968	2.694	0.135	2.586	1.666	12.270	0.010
**IIQ-7**								
		**B**	**Std. error**	**Beta**	**t**	**95%CI for B**		**p**
Model 1						LL	UL	
	Age	0.642	0.085	0.413	7.589	0.476	0.809	<0.001
	BMI	-0.339	0.373	-0.047	-0.909	-1.072	0.395	0.364
	Gravida	1.130	1.006	0.097	1.123	-0.850	3.109	0.262
	Parity	1.627	1.653	0.085	0.984	-1.626	4.880	0.326
	Sexual intercourse	-1.064	0.532	-0.103	-01.999	-2.111	-0.017	0.047
Model 2								
	Age	0.640	0.083	0.411	7.670	0.476	0.804	<0.001
	BMI	-0.320	0.367	-0.044	-0.871	-1.043	0.403	0.384
	Gravida	1.020	0.992	0.088	1.028	-0.932	2.972	0.305
	Parity	1.691	1.630	0.089	1.038	-1.516	4.898	0.300
	Sexual intercourse	-0.579	0.547	-0.056	-1.058	-1.655	0.498	0.291
	Group*	7.349	2.345	0.163	3.135	2.735	11.963	0.002

Note: 0=unmutilated, 1=mutilated.

* The "Group" parameter was included in the model with two groups coded as 0 for unmutilated and 1 for mutilated.

## DISSCUSSION

FGM has long been an area of research interest due to its profound social, cultural, and health implications. Our study is set against this backdrop, aiming to explore the relationship between FGM's different forms and UI symptoms in Sudan.

Out of 307 participants in our study, 52.44% reported undergoing FGM. This percentage echoes broader statistics within Sudan, where FGM remains a widespread and culturally deep-rooted practice^15^.

An elevated risk of health problems is just one of the many negative physical, mental, and emotional outcomes that may result. UI is one of the possible side effects of FGM. Problems with urinating and an increased chance of urine incontinence are among the potential consequences that can arise from the procedure's removal or injury to genital tissues. The level of incontinence that results from mutilation can differ in intensity based on the procedures that were carried out.

FGM procedures involve the removal or alteration of tissues around the urethra^16^. This can result in stenosis or occlusion of the urethral orifice, causing urinary flow obstruction. Various degrees of scar tissue occur in the external genitalia, depending on the severity of the excision. Scar tissue can be less elastic and flexible than normal tissue, potentially causing structural changes that affect the normal functioning of the urinary system. This scarring can contribute to UI^17^.

The removal of sensitive genital tissues during FGM procedures can lead to nerve damage^18^. Neuronal stimulation occurs in the medial paracentral lobule as a result of vulvar sensations. The anus and vagina are "mapped" together on the medial surface of the cortex, namely, between the central and precentral (marginalis) sulci. If there is a reorganization of neuronal input at the body surface, it may also lead to a reorganization of input to the somatosensory cortex^19^.

The connection between mutilation and UI can be comprehended by examining the existing body of literature on childhood sexual abuse and UI^20^. Several women who have been mutilated recall experiencing intense terror, excruciating pain, and a profound sense of helplessness. Mutilation, such as sexual abuse, is recognized to be a causative factor for post-traumatic stress disorder, somatization, depression, and anxiety^21,22^. In their study, Geynisman-Tan et al. attributed the increase in UI prevalence in patients with FGM to this relationship^7^.

Furthermore, Emanulle et al.'s systematic review strongly advocated for rigorous evidence in the form of randomized controlled trials to conclusively determine the urological complications associated with FGM, particularly Type III. Our study reaffirms this stance, indicating that infibulation (Type III FGM) is a significant contributor to UI, a sentiment our findings support. In particular, infibulation showed a strong association with UI symptoms^23^. Recognizing the study's limitations, particularly its sample size, is crucial.

We determined in this study that the more circumcision damages neighboring tissues and anatomical structures, the higher the rate of incontinence becomes.

The primary limitations of this study were the omission of urodynamic investigations and the reliance on urine analysis data instead of urine cultures for diagnosing urinary tract infections. These are notable flaws. Notwithstanding these constraints, the study offers an initial understanding of the impact of FGM on the occurrence of UI.

## CONCLUSION

Individuals who have been circumcised, particularly those with infibulation, are more likely to experience incontinence symptoms. Healthcare providers attending to patients with FGM/C should inquire about UI.
